# Characterization of the Soybean (*Glycine max*) Heavy-Metal-Associated Isoprenylated Plant Protein (*HIPP*) Gene Family in Response to Aluminum

**DOI:** 10.3390/plants14233582

**Published:** 2025-11-24

**Authors:** Jifu Li, Jiang Tian, Cuiyue Liang, Tianqi Wang, Xing Lu

**Affiliations:** 1 Root Biology Center, College of Natural Resourced Environment, South China Agricultural University, Guangzhou 510642, China; 2 Guangdong Engineering Technology Research Center of Low Carbon Agricultural Green Inputs, South China Agricultural University, Guangzhou 510642, China

**Keywords:** soybean, aluminum toxicity, transcriptome, heavy metal-associated isoprenylated plant proteins (HIPPs)

## Abstract

Heavy-metal-associated (HMA) isoprenylated plant proteins (HIPPs) play crucial roles in plant responses to biotic/abiotic stresses and heavy-metal homeostasis. However, the involvement of *HIPP* genes in the response of soybean (*Glycine max*) to aluminum (Al) toxicity remains unexplored. This study aimed to comprehensively characterize the *GmHIPP* gene family and investigate its role in Al toxicity. A total of 76 *GmHIPP* genes were identified in the soybean genome. Phylogenetic and synteny analyses revealed that HIPP evolution was highly conserved among soybean, Arabidopsis (*Arabidopsis thaliana*), and rice (*Oryza sativa*). Cis-element analysis indicated that *GmHIPP* genes might be involved in phytohormone response, abiotic and biotic stresses, and plant growth. RNA-seq analysis further revealed that the expression of 20 *GmHIPP*s was up-regulated, and three *GmHIPP*s were down-regulated under Al stress in roots. Among them, six genes (*GmHIPP9*/*13*/*29*/*43*/*58*/*73*) were highly induced by Al, with *GmHIPP29* exhibiting particularly high expression in root tips. Subcellular localization demonstrated that GmHIPP29 is a plasma membrane-localized protein. *GmHIPP*29-overexpression significantly increased Al accumulation in the cell sap of the transgenic soybean hairy root tips, leading to increased Al sensitivity. Collectively, these results demonstrate that *GmHIPP29* acts as a negative regulator of Al tolerance by promoting Al accumulation in soybean roots.

## 1. Introduction

Acid soils, encompassing over 50% of the world’s potentially arable lands, severely limit the production of staple crops by fixing essential nutrients and accumulation of toxic metals [1,2,3,4]. Among these constraints, aluminum (Al) toxicity, generally presenting in trivalent form (Al^3+^) in acid soils, emerges as particularly detrimental, limiting crop growth [1,5]. The phytotoxic effects of Al most acutely manifest as a rapid inhibition of root growth. This inhibition subsequently limits water and mineral nutrient uptake, ultimately decreasing crop yields [6,7]. Therefore, mitigating Al toxicity in plants is crucial for enhancing crop productivity and ensuring food security in acidic soils.

To adapt to Al toxicity, plants have evolved two primary detoxification mechanisms during evolution, including efflux detoxification and internal tolerance detoxification [1,4,8]. Both mechanisms heavily rely on transmembrane transporter pathways, which are mainly mediated by specialized transporter families such as the ATP-binding cassette (ABC) transporters [9], natural resistance-associated macrophage proteins (Nramp) [10,11], and heavy metal ATPase (HMA) [12]. Among them, Heavy-metal-associated isoprenylated plant proteins (HIPPs) are typical metal-binding metallochaperones. They are characterized by the presence of a heavy-metal-associated domain (HMA; PF00403) and a C-terminal isoprenylation motif (CaaX motif) [13]. Systematic genomic analyses have identified *HIPP* gene families in diverse plant species, including Arabidopsis (*Arabidopsis thaliana*) [14], rice (*Oryza sativa*) [15], tea plant (*Camellia sinensis*) [16], wheat (*Triticum aestivum*) [17], grapevine (*Vitis vinifera*) [18], tomato (*Solanum lycopersicum*) [19], sorghum (*Sorghum bicolor*) [20], and alfalfa (*Medicago sativa*) [21].

Functional studies reveal that *HIPPs* play multifaceted roles in plant growth and stress responses [14,18,22], with prominent functions in mediating plant tolerance to metal toxicity [13,23]. Specifically, HIPPs are known to bind, transport, accumulate, and detoxify various metal ions [13,23]. For example, in Arabidopsis, the *athipp 20/21/22* triple mutant exhibited enhanced cadmium (Cd) sensitivity and reduced Cd accumulation [24]. In rice, *OsHIPP29* contributes to Cd detoxification by reducing Cd accumulation, as evidenced by increased Cd sensitivity of knockout lines and enhanced tolerance of overexpression plants [25]. Conversely, overexpression of *OsHIPP24* leads to stunted growth and increased accumulation of Cu and Cd [26]. Furthermore, heterologous expression of *BcHIPP16* from pak choi (*Brassica campestris*) in yeast (*Saccharomyces cerevisiae*) increased the accumulation of Cu and Cd, although its overexpression in transgenic Arabidopsis showed no significant growth alteration under Cd or Cu exposure [27]. Despite these established roles in metal homeostasis, the potential involvement of HIPPs in plant responses to Al stress remains unexplored.

Soybean (*Glycine max*) is a globally cultivated legume, providing over 25% of the protein for human consumption and livestock feed [28,29,30]. However, its production in key regions, such as Brazil, Argentina, and Southern China, is severely constrained by acidic soils, where Al toxicity significantly inhibits growth and reduces yield [31]. Although several genes involved in Al detoxification have been identified in soybean [32], the genetic basis of its Al tolerance remains incompletely understood. In particular, the functions of *GmHIPP* genes in the Al stress response are largely unknown. Thus, this study was conducted to characterize the *GmHIPP* gene family and investigate its role in Al toxicity tolerance. We first performed a systematic genome-wide analysis to identify the *HIPP* family in soybean. The expression patterns of *GmHIPPs* under varying Al concentrations were then analyzed using transcriptomic data. Finally, we functionally validated the role of *GmHIPP29* by employing a transgenic hairy root system with both overexpression and silencing constructs. Our findings provide important insights into the roles of *GmHIPPs* in Al accumulation and tolerance.

## 2. Results

### 2.1. Identification and Chromosomal Distribution of GmHIPP Genes in the Soybean Genome

To systematically identify the *GmHIPP* genes in the soybean genome, we performed a BLASTp (version 2.14.0) search of the soybean proteome database using the full-length of the HAM domain as a query. This analysis revealed 76 *GmHIPP* genes (Table S2), encoding proteins ranging from 99 aa (*GmHIPP*66) to 560 aa (*GmHIPP*35) in length. The predicted isoelectric points (pI) of *GmHIPPs* ranged from 4.96 (*GmHIPP*3) to 9.79 (*GmHIPP*69), suggesting functional diversity among these proteins.

**Figure 1:**
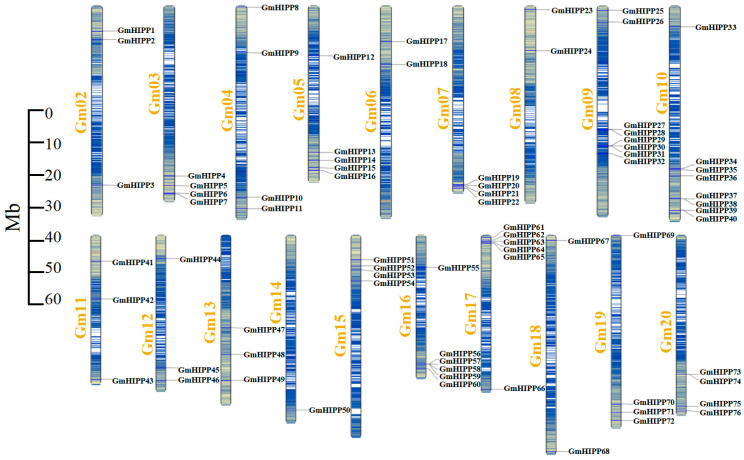
Chromosome distribution mapping of GmHIPP family in soybean. Scale indicates chromosome length, and chromosome colors blue to orange indicate gene density.

Based on the *Glycine max Wm82.a4.v1* genome information of soybean. The results of chromosomal distribution analyses showed that the 76 *GmHIPP* genes in soybean were irregularly distributed on 19 of the 20 chromosomes of soybean, with the numbers of *HIPP* members varying from one to eight (Figure 1). Among them, Chromosome 9 and 10 contained the largest number of *GmHIPP* genes (eight), followed by Chromosome 16 and 17 with six *GmHIPP* members. Chromosome 3/7/14/15/19/20 each had four *GmHIPP* members, while chromosome 14 contained only one *GmHIPP* gene. Interestingly, no gene was found on chromosome 1. In addition, three tandemly duplicated gene pairs were distributed on chromosomes 9 and 16, respectively.

### 2.2. Phylogenetic Tree, Domains, Conserved Motifs, and Gene Structure of GmHIPP Family

**Figure 2:**
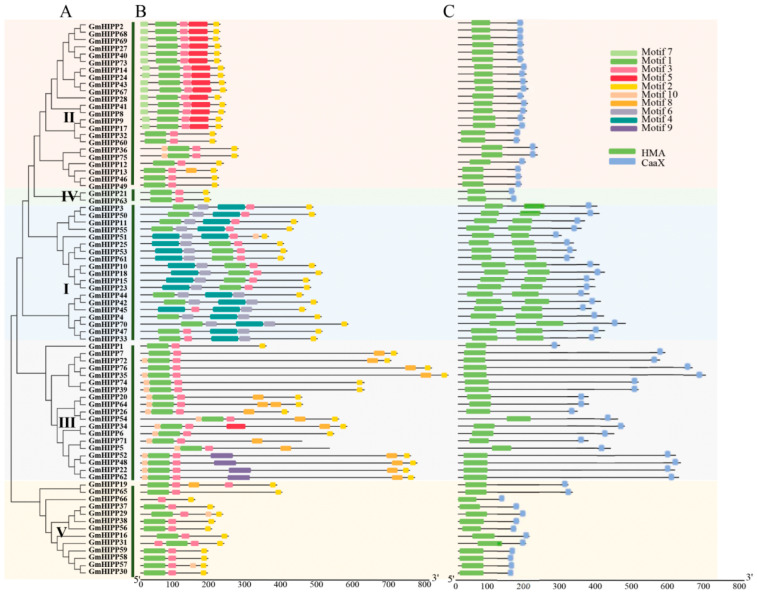
Phylogenetic relationship, protein structure, and conserved domains of GmHIPP family members. (**A**) Phylogenetic relationship of the identified GmHIPPs in the soybean genome. An unrooted Neighbor-Joining (NJ) tree was constructed using MEGA-X. A bootstrap test with 1000 replicates was performed to assess branch support. (**B**) Distribution of the conserved motifs in GmHIPP proteins. Ten conserved motifs are marked with different colored boxes. (**C**) The distribution of conserved domains in GmHIPP proteins. Green and blue boxes denote conserved HMA and CaaX motifs, respectively.

To determine the homology relationships among the identified soybean HIPP family members, a phylogenetic analysis was conducted using the aligned sequences of 76 HIPP proteins (Figure 2A). The topology of the resulting phylogenetic tree indicated that 76 GmHIPP proteins could be divided into five groups: Group I to V. Group I contains 19 members, Group II harboreds 23 members, Group III contains 20 members, Group V includes 13 members, and Group IV only contains GmHIPP21 and GmHIPP63.

To unravel the differences among GmHIPP family members, we analyzed their conserved motifs, structural domains, and gene structure. A total of 10 conserved motifs were identified using the MEME website (Figure 2B), and similar motif distribution pattern was found in each group. Moreover motif1/2/3, as the core domains, were commonly distributed across all GmHIPP family members. Specifically, motif 4 and motifs 6 were exclusively confined to Group I, while motif 5 was unique to Group II. Interestingly, two domains, HMA domain and CaaX domain were encoded by GmHIPP family members. Particularly, HMA domain and CaaX domain were identified in all GmHIPPs (Figure 2C).

Furthermore, the exon-intron structure analysis showed that 75% (57 *GmHIPPs*) of the *GmHIPP* family genes processed 1–3 introns, 18 *GmHIPPs* contained four introns, and only one *GmHIPP* gene (*GmHIPP66*) lacked an intron. Additionally, the members within each group possessed similar exon-intron structures (Figure S1). For example, the members of group IV only contained two exons, while group II members had 2–4 exons (Figure S1).

### 2.3. Phylogenetic Analysis of HIPP Proteins from Three Species

**Figure 3:**
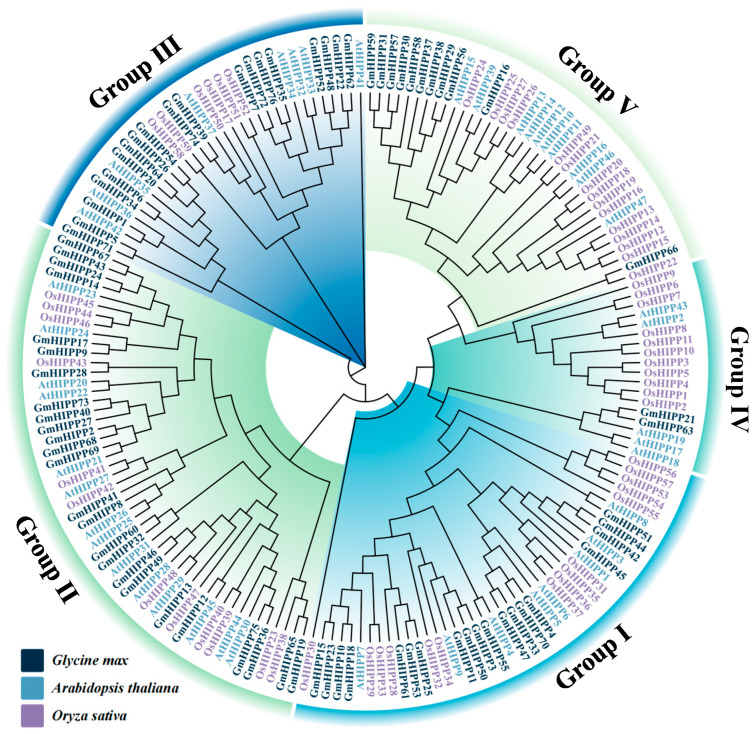
Phylogenetic relationships of HIPP proteins from soybean (*Glycine max*), Arabidopsis (*Arabidopsis thaliana*), and rice (*Oryza sativa*). An unrooted Neighbor-Joining (NJ) tree was constructed using MEGA-X. A bootstrap test with 1000 replicates was performed to assess branch support. Five distinct clades (Group I–V) of HIPP proteins are color-coded. The HIPP proteins of soybean, Arabidopsis, and rice are labeled black, blue, and purple, respectively.

To further explore the evolutionary relationship among *HIPP* genes in soybean, a phylogenetic tree was constructed by an unrooted neighbor-joining (NJ) method of 45 *Arabidopsis*, 59 *rice*, and 76 *soybean* HIPPs (Figure 3). The *HIPPs* proteins were separated by a phylogenetic analysis into five groups (Group I to V). Group II, the largest group, contained 51 *HIPP* proteins, including 25 from soybean, 14 from *Arabidopsis*, and 12 from *rice*. Followed by Group I, which harbored 39 members with 19 GmHIPP proteins, 12 OsHIPPs, and eight AtHIPPs. Group V included 36 members, with 11 GmHIPP proteins, 15 OsHIPPs, and 10 AtHIPPs. Group III comprised 33 members, including 19 GmHIPPs, eight AtHIPPs, and six OsHIPPs. In contrast, Group IV only harbored 2 *GmHIPP* proteins. Overall, groups I, II, and III contained more *HIPP* proteins in *soybean* compared to those in *Arabidopsis* and *rice*, suggesting that after the core eudicot divergence, a species-specific expansion of *GmHIPP* proteins occurred in soybean.

### 2.4. Evolutionary and Synteny Analysis of HIPP Gene in Soybean

**Figure 4:**
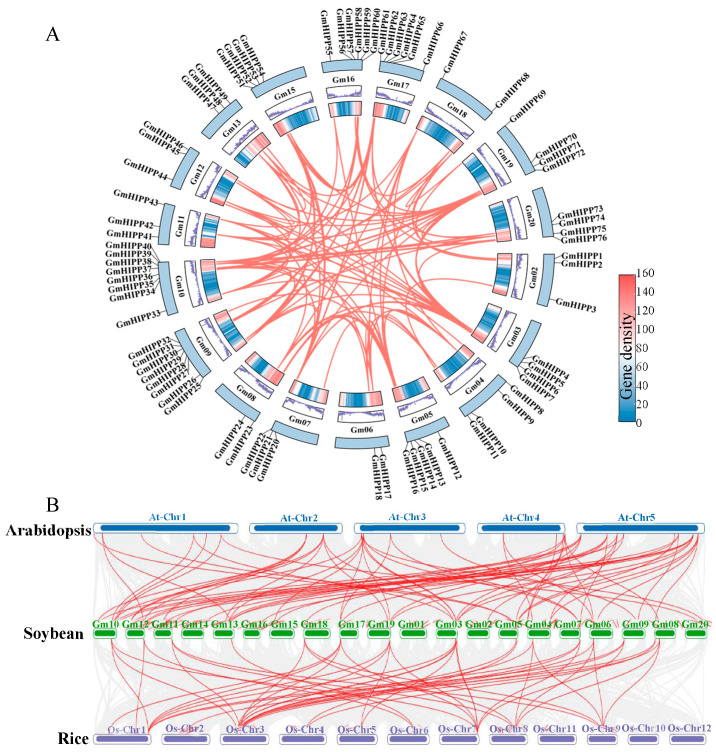
Duplication and synteny analysis of *HIPP* genes. (**A**) Collinearity analysis of *HIPP* in soybean chromosomes. The outermost ring displays the 20 chromosomes of the soybean genome (Gm01–Gm20). The locations of the 76 *GmHIPP* genes are marked along the chromosomes. The heatmaps in the inner rings indicate gene density, with the gradient ranging from low (blue) to high (red). The red lines within the circle represent segmental duplication events between *GmHIPP* genes, illustrating their collinear relationships across the genome. (**B**) Collinearity analysis of *HIPP* genes in soybean, rice, and Arabidopsis. Gray lines indicate all syntenic blocks in the genome, red lines represent homologous *HIPP* gene pairs.

To investigate the evolutionary dynamics of *GmHIPP* genes, we identified segmental duplications through collinearity analysis. The intraspecific synteny analysis showed tandem or segmental duplication events among *GmHIPP* members in the soybean genome, and 111 pairs of segmental-duplicated genes were found on soybean chromosomes, and segmental duplication events were distributed across multiple chromosomes, particularly chromosomes 9, 10, 16, and 20 (Figure 4A). Notably, chromosome 9 contained the most segmental duplications, suggesting its pivotal role in the expansion of the *HIPP*s. Furthermore, we selected the model plant Arabidopsis and rice as the reference genomes and generated genomic collinearity plots between *GmHIPP, AtHIPP*, and *OsHIPP* genes. The analysis identified 64 syntenic pairs between *GmHIPP* genes and *AtHIPP* genes, and 45 syntenic pairs between *GmHIPP* genes and *OsHIPP* genes, suggesting that the *GmHIPP* genes are more closely evolutionarily related to the *AtHIPP* genes (Figure 4B).

### 2.5. Analysis of the Promoter Region of the GmHIPP Gene Family

**Figure 5:**
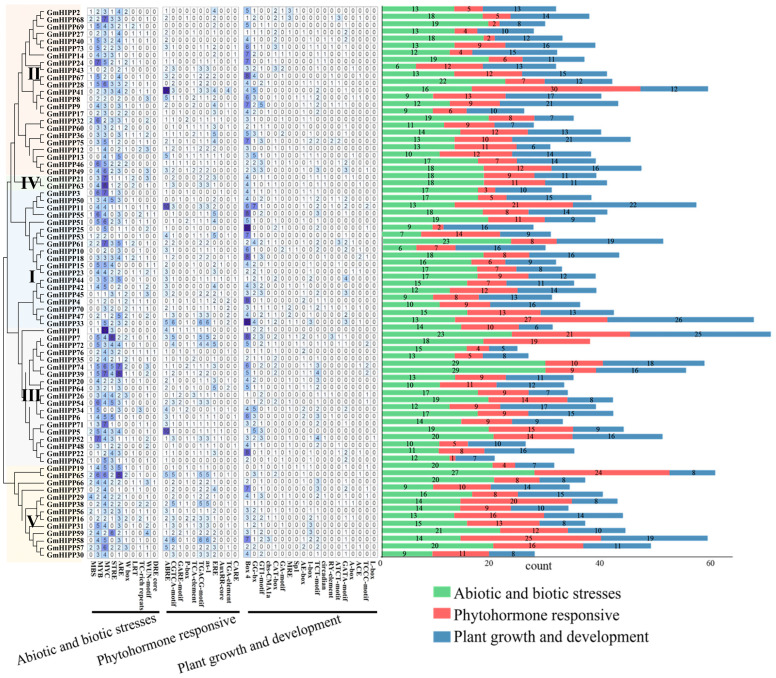
Analysis of cis-acting elements in the promoter region of *GmHIPP* genes. The 40 cis-elements were categorized into three main groups, which are separated by horizontal lines. The numbers in the grid cells represent the count of cis-elements, and color gradient from white to dark blue indicates an increasing number of cis-elements from fewer to more.

To elucidate the potential biological functions of *GmHIPP* genes in soybean growth, development, and stress responses, we analyzed the 2.0 kb sequence upstream of the *GmHIPP* genes and identified cis-acting elements consistent with the gene transcription direction. A total of 40 cis-elements were identified and classified into three functional categories, including abiotic and biotic stress, phytohormone-responsive, and plant growth and development (Figure 5). Among them, the largest part of the cis-elements, belonging to the abiotic and biotic stress category, is associated with three general stress-responsive motifs: the MYB (CCAAT box), MYC (CACATG box), and ARE binding sites. Among the abiotic and biotic stress, 92% of the *GmHIPP* genes contained MYB elements, 98.7% contained MYC elements, and 94.7% contained ARE elements.

Phytohormone response elements include abscisic acid response element ABRE and CGTCA-motif, auxin response elements AuxRR-core and TGA-element, gibberellin response related elements P-box, ERE and GARE-motif, salicylic acid response element TCA-element, MeJA response elements, TGACG-motif. Among the phytohormone response elements, 78.9% of the *GmHIPP* genes contained abscisic acid response element ABRE, and 80.3% harbored gibberellin response related elements ERE. In the plant growth and development group, a total of 18 cis-elements were identified. Remarkably, among these, 94.7% of the *GmHIPP* genes contained Box 4 elements (Figure 5). These data suggest the potential role of *GmHIPP* gene in plant growth, development, and environmental stress responses.

### 2.6. Transcriptomic Changes in Soybean Roots Exposed to Al Toxicity

**Figure 6:**
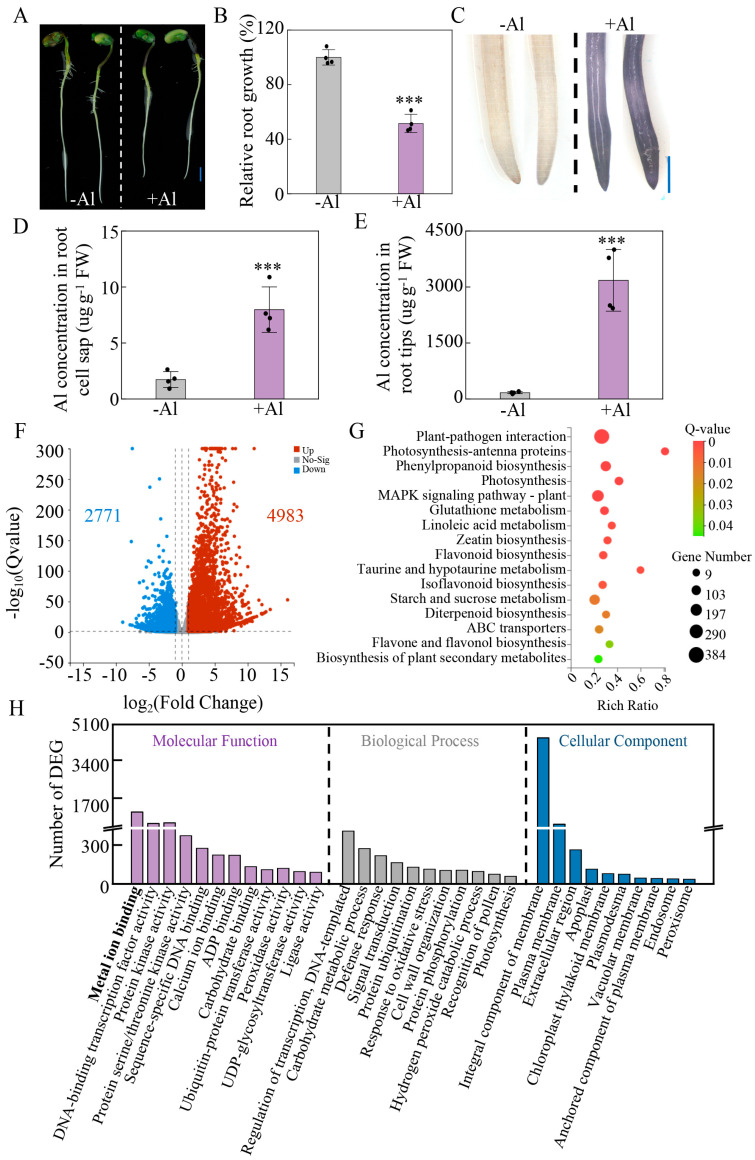
RNA-Seq analysis of Al-responsive genes in root tips of soybean. (**A**) Root growth performance. Bar = 2 cm. (**B**) Relative root growth. (**C**) Hematoxylin staining analysis. Bar = 0.5 mm. (**D**) Al concentration in root cell sap. (**E**) Al concentration in root tips. (**F**) Volcano plot of differentially expressed genes (DEGs) in soybean root tips. (**G**) KEGG pathway analysis of DEGs in soybean roots. (**H**) Gene Ontology (GO) analysis of DEGs in soybean roots. Root tips of the soybean with treated with 0 (−Al) and 50 (+Al) μM AlCl_3_ for 24 h and used for RNA-seq analysis. Data are presented as mean ± standard error (SE) (*n* = 4). Asterisks denote significant differences between the +Al treatment and −Al control using Student’s *t*-test. ***, *p* < 0.001.

To explore the growth response of soybean to Al toxicity, hydroponic experiment with two Al concentrations (−Al, 0 μM; +Al, 50 μM) was conducted. +Al treatment significantly inhibited root growth (Figure 6A), represented by a 48.4% reduction in relative root growth compared to the control (Figure 6B). Hematoxylin staining revealed pronounced Al accumulation in root tips, with staining intensity increasing in a dose-dependent manner (Figure 6C). Furthermore, results showed that Al concentrations in root cell sap and root tips exposed to 50 μM Al were 4.7-fold and 18.9-fold greater than those in the control, respectively (Figure 6D,E). These findings demonstrate that Al toxicity severely impairs soybean root elongation and leads to preferential Al accumulation in root tips.

To elucidate the molecular mechanisms underlying Al toxicity responses, we conducted transcriptomic analysis on roots of soybean. Under the criterion of *q* < 0.05 and |log_2_Fold Change| ≥ 1, a total of 7754 genes were identified as differentially expressed genes (DEGs) in soybean roots between the two Al treatments. Totally, 4983 genes were up-regulated and 2771 genes were down-regulated by Al toxicity (Figure 6F). KEGG pathway enrichment revealed that DEGs were predominantly associated with 16 metabolic pathways. Among them, 384 DEGs were closely related to plant–pathogen interaction and 103 DEGs were closely related to phenylpropanoid biosynthesis (Figure 6G). Gene Ontology (GO) classification analysis showed that the Al-responsive genes can be divided into three categories, including molecular function (MF), biological process (BP), and cellular component (CC) (Figure 6H). All the three categories can be classified into 33 subcategories, including 12 terms in MF, 11 terms in BP, and 10 terms in CC. Among the MF group, the significantly enriched subcategories included metal ion binding, DNA-binding transcription factor activity, protein kinase activity, calcium ion binding, and peroxidase activity (Figure 6H). In the BP group, the five most dominant terms contained regulation of transcription, carbohydrate metabolic process, defense response, signal transduction, and response to oxidative stress (Figure 6H). For the CC group, integral component of membrane, plasma membrane, extracellular region, apoplast, and chloroplast thylakoid membrane were the main subcategories (Figure 6H). To validate the transcriptomic results, qRT-PCR analysis was performed on 15 genes (*PME1*, *SOD1*/*SOD2*, *POD1*-*POD6*, *GST1*-*GST6*) using gene-specific primers. The results demonstrated similar trends to the transcriptomic data (Figure S2), confirming the reliability of transcript abundance estimates.

### 2.7. Identification of Al-Responsive Genes Related to Heavy Metal-Associated Isoprenylated Plant Protein (HIPP)

**Figure 7:**
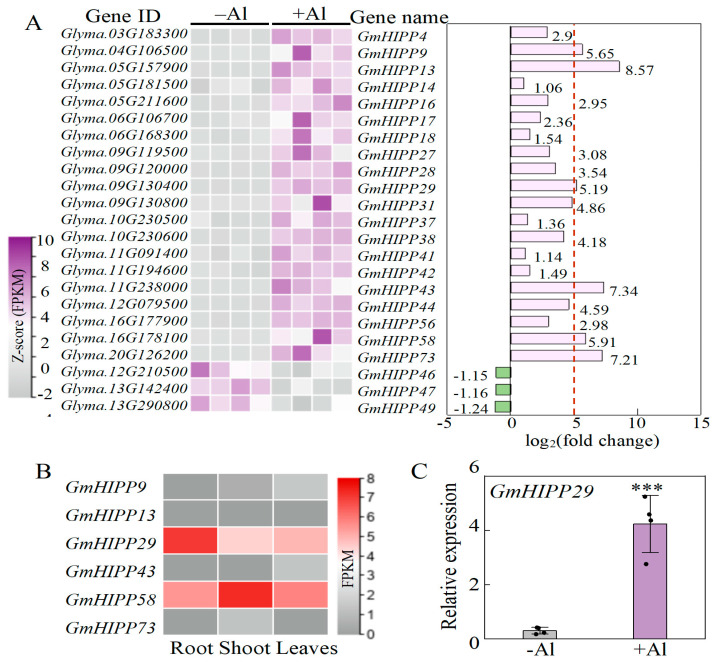
Expression patterns of *GmHIPPs* in response to Al toxicity. (**A**) Expression patterns of *GmHIPPs* in root tips of soybean. The FPKM data were normalized by Z-score. The bar chart shows the log_2_(fold change) values; with the red dashed line representing stands for log_2_(Fold Change) = 5. (**B**) Expression patterns of *GmHIPP9*/*13*/*29*/*43*/*58*/*73* (log_2_FC > 5) in different tissues of soybean. (**C**) Expression of *GmHIPP29* in root tips with 0 (−Al) and 50 (+Al) μM Al treatments. Data are presented as mean ± standard error (SE) (*n* = 4). Asterisk indicates significant differences between the +Al treatment and −Al control using Student’s *t*-test. ***, *p* < 0.001.

GO analysis identified 1079 DEGs associated with metal ion binding (Table S3). These DEGs encoded heavy metal-associated isoprenylated plant protein (*HIPP*), copper transport protein ATOX1-like (*ATX-like*), heavy-metal ATPases (*HMA*), metal ion binding protein (*MBP*), heavy metal-associated plant protein (*HPP*), and copper chaperone (CCH). Notably, 23 genes were related to heavy metal-associated isoprenylated plant protein (*HIPP*), including 20 up-regulated and three down-regulated genes (Figure 7A). Among them, the expressions of *GmHIPP9*, *GmHIPP13*, *GmHIPP29*, *GmHIPP43*, *GmHIPP58*, and *GmHIPP73* were significantly up-regulated by more than 10-fold (Figure 7A).

Furthermore, the expression patterns of six *GmHIPP* genes with high Al responsiveness were analyzed across leaves, shoots, and roots (Figure 7B). Strikingly, *GmHIPP29* showed predominant root-specific expression compared to its expression in leaves and shoots, suggesting a potential role in root responses to Al stress. The qRT-PCR analysis confirmed that *GmHIPP29* expression in root tips increased by 127.1% under +Al treatment compared to the control (Figure 7C). Given its strong Al-responsive induction and root-enriched expression, *GmHIPP29* was selected for further functional characterization.

### 2.8. Subcellular Localization of GmHIPP29

**Figure 8:**
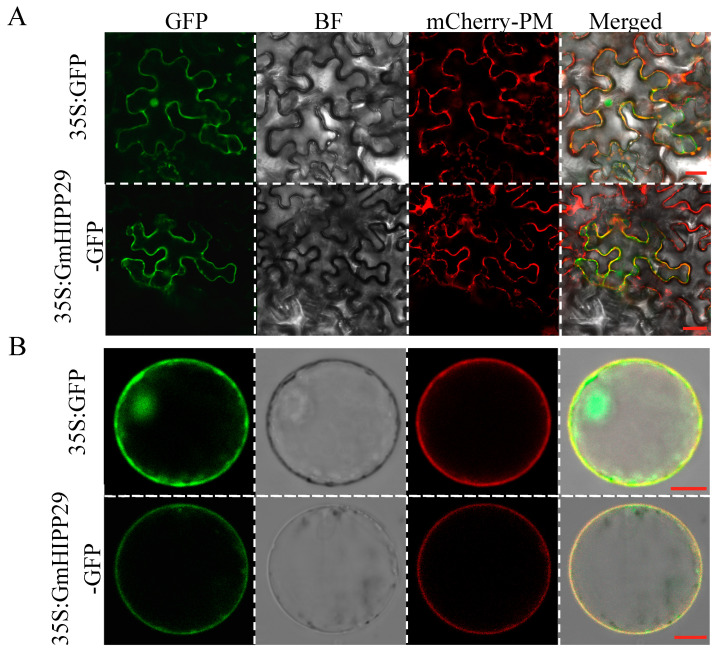
Subcellular localization analysis of GmHIPP29. (**A**) Subcellular localization in tobacco leaves. Bars = 20 μm. (**B**) Subcellular localization in rice protoplasts. Bars = 10 μm. The green fluorescence derived from either 35S:GFP and 35S:GFP-GmHIPP29, and the red fluorescence derived from 35S:AtPIP2A-mCherry were observed by the confocal microscope.

Subcellular localization assays in the tobacco leaf epidermic cells demonstrated that the green fluorescence from 35S:GmHIPP29-GFP is predominantly localized to the plasma membrane. In contrast, green fluorescence from the control 35S:GFP was detected in the plasma membrane, nucleus, and cytoplasm (Figure 8A). Similarly, the microscopy analysis revealed that GmHIPP29-GFP was specifically localized on the plasma membrane in rice protoplasts. These results consistently indicate that GmHIPP29 is a plasma membrane associated protein (Figure 8B).

### 2.9. Alternative Expression of GmHIPP29 Affects Soybean Hairy Roots Growth in Response to Al Toxicity

**Figure 9:**
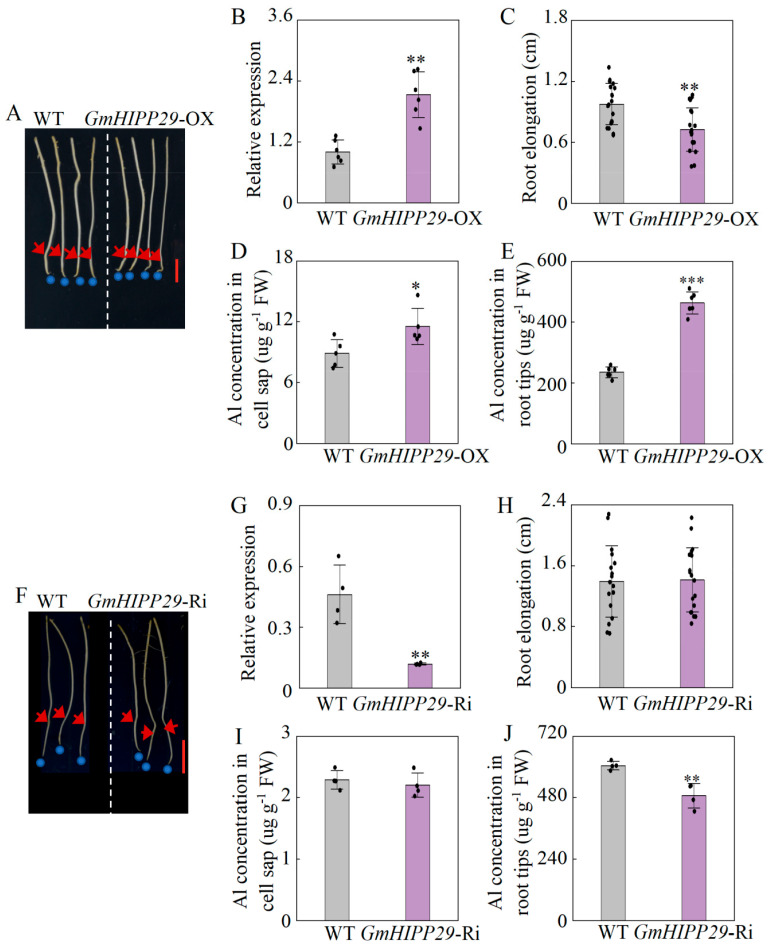
Verification of transgenic soybean hair roots and the function of *GmHIPP29* in Al tolerance. (**A**) Phenotypes of *GmHIPP29*-OX hairy roots under Al treatment. Bar = 1 cm. (**B**) Relative expression levels of *GmHIPP29*. WT represents the empty vector (*pTF101s*) control line and *GmHIPP29*-OX represents the overexpression line. (**C**) Root elongation under Al treatment for WT/*GmHIPP29*-OX lines. (**D**) Al concentration in the root tip cell sap of WT/*GmHIPP29*-OX lines. (**E**) Al concentration in the root tips of WT/*GmHIPP29*-OX lines. (**F**) Phenotypes of *GmHIPP29*-Ri hairy roots under Al treatment. Bar = 1 cm. (**G**) Relative expression levels of *GmHIPP29*. WT represents the empty vector (*pFGC5941*) control line, *GmHIPP29*-Ri represents the RNA interference line. (**H**) Root elongation under Al treatment for WT/*GmHIPP29*-Ri lines. (**I**) Al concentration in the root tip cell sap of WT/*GmHIPP29*-Ri. (**J**) Al concentration in the root tips of WT/*GmHIPP29*-Ri. For Al treatment, the uniform soybean hairy roots were subjected to 0 and 50 µM AlCl_3_ in 0.5 mM CaCl_2_ (pH 4.5) solution for 24 h. OX, overexpression; Ri, RNA interference. Data are presented as mean ± standard error (SE) (*n* = 6 for (**B**–**E**) and *n* = 4 for (**G**–**J**)). Asterisks indicate significant differences between the WT and *GmHIPP29* OX/Ri lines under Al treatment using Student’s *t*-test. *, *p* < 0.05. **, *p* < 0.01. ***, *p* < 0.001.

To assess the role of *GmHIPP29* in the regulation of Al acquisition, we generated transgenic soybean hairy roots with *GmHIPP29* overexpression (*GmHIPP29*-OX) and RNA interference (*GmHIPP29*-RNAi) (Figure 9). Transgenic lines expressing empty vector (either *pTF101s* or *pFGC5941*) served as the corresponding wild-type (WT) controls. The enhanced expression of *GmHIPP29* was verified through qRT-PCR analysis, which showed a 2.12-fold increase in the transgenic overexpression soybean hair roots (Figure 9B). Under +Al conditions, overexpression of *GmHIPP29* inhibited hairy root growth, as reflected by a 25.5% decrease in root elongation in the overexpression lines compared to the controls (Figure 9C). Moreover, *GmHIPP29* overexpression lines displayed a 30% and a 96.9% increase in the Al concentrations of root tips cell sap and root tips, respectively, compared to the WT controls in the presence of external Al (Figure 9D,E).

Conversely, qRT-PCR analysis of transgenic roots confirmed that the expression level in the knockdown lines (*GmHIPP29-*RNAi) was 2.92-fold lower than that in the WT controls during the application of external Al (Figure 9G). However, no significant differences were observed in root elongation and Al concentration in root tips cell sap between WT and *GmHIPP29-*RNAi lines under Al treatments (Figure 9H,I). Additionally, *GmHIPP29-*RNAi lines displayed a 19.5% reduction in root tips Al concentrations compared to the WT controls when subjected to external Al stress (Figure 9J). These results indicate that the expression of *GmHIPP29* significantly regulates the physiological response to Al tolerance in soybean.

## 3. Discussion

Soybean is an important legume crop serving as one of the major sources of protein and edible oil. However, its production and quality are severely constrained by Al toxicity in acid soils. The Al-resistance mechanisms in soybean have been widely characterized [32]. These strategies include, but are not limited to, enhanced organic acid exudation [33,34], cell wall modification [35,36], Al exclusion by border cell formation [37,38], and ROS scavenge [39,40]. These physiological and biochemical changes are regulated by various transcription factors, such as C2H2 zinc finger type transcription factor (STOP), WRKY, and NAC families [41,42,43]. Although a few Al-tolerant genes involved in exclusion mechanisms and internal tolerance mechanisms have been functionally characterized [32], gene resources for Al tolerance in soybean remain insufficient compared to the model plants like Arabidopsis and rice. 

### 3.1. Structure Characteristics of GmHIPP

The *HIPP* family genes play important roles in plant responses to various abiotic stresses [13,23], as reported in Arabidopsis [24], rice [15], wheat [17], tomato [19], sorghum [20], and alfalfa [21]. However, a comprehensive characterization of *GmHIPPs* in soybean has been lacking. Our systematic analysis identified 76 *GmHIPP* genes in soybean; these genes exhibit conserved evolutionary patterns while maintaining certain lineage-specific features (Figure 2 and Table S2). Furthermore, the distribution of these genes on chromosomes and the evidence of segmental duplication events highlight the expansion of *HIPP* family in the soybean genome (Figure 1 and Figure 4). Such expansion is known to assist plants in adapting to various environmental stresses [44].

The crucial roles of HIPPs in heavy metal stress responses primarily involve regulating uptake, translocation, and homeostasis of metal ions [13]. These functional roles are largely attributed to their conserved structural features: a HMA domain for metal binding, and a C-terminal isoprenylation motif that facilitates membrane localization [23]. Phylogenetic analysis revealed that proteins within the same clade are likely to share similar biological functions. For instance, AtHIPP20/21/22 in Arabidopsis and their ortholog OsHIPP42 in rice, which cluster within group II (Figure 3), have both been demonstrated to confer Cd tolerance by binding heavy metals and reducing cellular Cd accumulation [15,24]. Furthermore, OsHIPP16 and OsHIPP24 in group III also involve in Cd accumulation [15,26]. In our analysis, specific GmHIPP members showed close evolutionary relationships with these characterized proteins: Group II members GmHIPP2/27/40/68/69/73 clustered with AtHIPP20/21/22, while Group III members GmHIPP16/29/56 were more closely related to OsHIPP24 (Figure 3). These results suggest that the GmHIPP family likely functions in mediating metal ion uptake.

### 3.2. GmHIPP Genes in Response to Al Toxicity

The transcriptional regulation of *HIPPs* by Al toxicity has not been well understood, although the expression patterns of plant *HIPPs* have been well characterized in response to Cd, manganese (Mn), and Cu stresses [15,27,45]. Through RNA-Seq analysis, we found that 20 *GmHIPP*s were up-regulated and three *GmHIPP*s were down-regulated in response to Al (Figure 7A), suggesting their potential involvement in Al tolerance in soybean. Six genes (*GmHIPP9*/*13*/*29*/*43*/*58*/*73*) were notable. Given that Al-responsive genes are often preferentially expressed in root tips [33,41,46], we performed tissue-specific expression analysis. The results showed that *GmHIPP29* exhibited the highest transcript levels in roots, with a significant induction in root tips under Al stress (Figure 7B,C). This expression pattern strongly suggests a vital role for *GmHIPP29* in the soybean Al response mechanism.

In functional validation, overexpression of *GmHIPP29* significantly reduced root elongation and increased Al accumulation in hairy roots (Figure 9C–E). However, suppression of *GmHIPP29* did not noticeably affect root growth (Figure 9H). This discrepancy might be explained by functional redundancy among *HIPP* genes. Many *HIPP* family members are likely co-expressed and may have overlapping roles in metal binding and trafficking [13]. Consequently, the partial knockdown of a single *GmHIPP* gene could be functionally compensated for by other members, thereby mitigating observable phenotypic effects. Such compensation is well-documented in polyploid genomes like soybean [47,48]. In addition, the relatively weak phenotypic alterations in the RNAi lines can be attributed to random T-DNA integration, copy numbers, and silencing potential per root, directly affecting transgene expression and influencing root growth prior to transgene effects, an inherent characteristic of the *Agrobacterium rhizogenes*-mediated root transformation system [49,50].

The regulatory roles of HIPP proteins in plant metal stress are multifaceted, with individual members acting as either positive or negative regulators [20,25,26,27,51]. In this study, *GmHIPP29* was identified as a negative regulator of Al tolerance. This finding mirrors the reported roles of *SbHIPP40* in Cd stress and *OsHIPP17/24* in Cu stress [20,26,51]. For example, rice plants overexpressing *SbHIPP40* demonstrated inhibited root growth and increased Cd accumulation when exposed to Cd [20]. Similarly, *OsHIPP17* and *OsHIPP24* in rice roots were found to be up-regulated by Cu stress. Overexpression of *OsHIPP17* in Arabidopsis and *OsHIPP24* in rice increased root Cu uptake and inhibited plant growth [26,51]. In this study, GmHIPP29 is localized to the plasma membrane (Figure 8), which is consistent with the characteristics of BcHIPP16, OsHIPP24, and OsHIPP29 [25,26,27]. These homologs have been implicated in mediating the uptake and transport of multiple metal ions, including Cd, Fe, Mn, and Cu, through their plasma membrane localization [25,26,27]. Hence, GmHIPP29 may function in Al ion uptake in soybean.

### 3.3. The Potential Mechanism of GmHIPP29 in Regulating Al Tolerance in Soybean

The *HIPP* family is considered to positively regulate heavy metal tolerance in some plants. However, our study reveals for the first time that *GmHIPP29* acts as a negative regulator under Al stress. This unexpected finding can be attributed to the distinct toxicity mechanisms of different metals. Previous research on HIPPs has primarily focused on their roles in mitigating the toxicity of Cd and Cu [25,26], which largely disrupt intracellular redox homeostasis and displace essential metal cofactors [23]. In contrast, Al toxicity primarily involves physical and surface-level interactions, such as cell wall hardening, disruption of plasma membrane integrity, and induction of reactive oxygen species (ROS) [4]. Consequently, HIPP proteins specialized for chelating Cd or Cu may not be functionally equipped to manage Al stress effectively. Instead, their interaction with Al might inadvertently interfere with critical detoxification pathways, potentially exacerbating Al toxicity in plants.

We identified GmHIPP29 as a plasma membrane protein that increases Al uptake and thereby negatively impacts Al tolerance in soybean hairy roots (Figure 9). However, the precise molecular mechanism remains elusive. Effective Al tolerance in plants generally requires the coordinated action of plasma membrane transporters and endomembrane transporters to sequester cytoplasmic Al into vacuoles [9,52,53]. We hypothesize that GmHIPP29 may facilitate Al entry into the cytosol and potentially disrupt the vacuolar sequestration pathway. This is supported by the observed increase in cytosolic Al levels in *GmHIPP29*-overexpressing lines (Figure 9D). However, further research is needed to determine whether *GmHIPP29* expression affects the function of known vacuolar Al transporter, such as the *Nramp* aluminum transporter (*NRAT*) family [10].

Additionally, GmHIPP29 may interfere with the long-distance translocation of Al. In Arabidopsis, the transporter Aluminum Sensitive1 (ALS1) plays a crucial role in both vacuolar sequestration of Al in root cells and the loading of Al into the xylem for transport to shoots [52,53]. If GmHIPP29 binds Al with high affinity in the cytosol of root tips, it might hinder the transfer of Al to ALS1-like transporters. This would result in the harmful accumulation of Al in the root apex, consistent with our data showing increased Al concentration in root tips and reduced root elongation. Future research should explore the functional relationship between GmHIPP29 and ALS1-like transporters to better understand Al transport and detoxification in soybeans.

Interestingly, beyond directly mediating metal uptake, *HIPP* genes can also regulate the expression of metal transporter genes, thereby indirectly affecting metal ion homeostasis [54,55]. For example, the *oshipp17* mutant exhibited increased expression of Cd transporters *OsNRAMP1* and *OsNRAMP5*, leading to Cd accumulation in rice roots [54]. Similarly, overexpression of *ApHIPP26* in Arabidopsis enhanced the expression of *AtNRAMP6* and *AtNRAMP3* related to the absorption and transport of heavy metals and resulted in Cd accumulation [55]. However, whether *GmHIPP29* mediates Al homeostasis in soybean through affecting the expression of Al transporter genes remains to be experimentally verified.

## 4. Materials and Methods

### 4.1. Identification of GmHIPP Family Genes in Soybean

The genome sequence of soybean was downloaded from the phytozome database (https://phytozome.jgi.doe.gov (accessed on 26 July 2024)) (*Glycine max* Wm82.a4.v1). The hidden Markov model (HMM) profile of the HMA domain (PF00403) was used as a query to scan the soybean genome. Redundant and short sequences were removed based on their physical localizations. All candidates were further verified using the Pfam and Simple Modular Architecture Research Tool (SMART) databases (https://smart.embl.de, accessed on 26 July 2024). Finally, candidate genes with the HMA domain were considered to be *GmHIPP* family genes.

### 4.2. Chromosome Distribution and Protein Structure Analysis of the HIPP Gene Family

Chromosomal information of *GmHIPP* genes was obtained by aligning cDNA sequences to the genomic sequence using BLAST (version 2.14.0). Chromosomal mapping was visualized using Tbtools [56]. Gene structures of the *GmHIPPs* were annotated using genome GFF files from Phytozome and analyzed with TBtools (version 2.056). The MEME website (https://meme-suite.org/meme/ (accessed on 29 July 2024)) was used to predict the conserved motif of HIPP proteins, with the number of motifs set to 10. The conserved domain were analyzed using the Batch Web CD-Search Tool (https://www.ncbi.nlm.nih.gov/Structure/cdd/wrpsb.cgi (accessed on 29 July 2024)). An integrated visualization of the phylogenetic tree, conserved motifs, and protein domains was generated using the TBtools [56]. The corresponding evolutionary trees were constructed by MEGA X as described above. The formation of the protein structure was constructed using Tbtools [56].

### 4.3. Phylogenetic Analysis of the HIPP Gene Family

The GmHIPPs protein sequences for the phylogenetic tree were obtained from the phytozome database (https://phytozome.jgi.doe.gov (accessed on 26 July 2024)), AtHIPPs and OsHIPP protein sequences according to De Abreu-Neto et al. [23]. HIPP amino acid sequences were subjected to multiple sequence alignments using Clustal W with default parameters. The evolutionary relationships were analyzed using MEGA X [57]. A phylogenetic tree was constructed with the value of the 1000 bootstrap replications using the neighbor-joining (NJ) method. The phylogenetic tree visualization was performed using the online tool Evolview (www.evolgenius.info/evolview/#/ (accessed on 7 August 2024)) [58].

### 4.4. Collinearity Analysis

Microsynteny relationship of *HIPP* genes among soybean, Arabidopsis, and rice were analyzed using the Dual Systeny Plotter in TBtools (https://github.com/CJ-Chen/TBtools (accessed on 8 August 2024)) [56]. The sequence similarity of *GmHIPP* genes was visualized using the Circoletto online tool (https://bat.infspire.org/circoletto/ (accessed on 10 August 2024)) [59].

### 4.5. Cis-Regulatory Element Analysis in the Promoter Regions of HIPP Genes in Soybean

The 2000 bp upstream sequences of the *HIPP*-encoding DNA were extracted from the soybean genome data using the TBtools. Cis-acting elements were identified using the PlantCARE (https://bioinformatics.psb.ugent.be/webtools/plantcare/html/ (accessed on 12 August 2024)), with exclusion of core promoter elements and functionally ambiguous motifs. Forty cis-elements were retained for visualization using TBtools [56].

### 4.6. Plant Growth Conditions and Al Treatment

The hydroponic experiment was conducted in the greenhouse of Root Biology Center in South China Agricultural University. Soybean (*Glycine max* cv. YC03-3) seeds were surface-sterilized with 10% (*v*/*v*) NaClO, germinated in paper towels saturated with 1/4 Hoagland nutrient solution (pH 5.8), and grown in controlled-environment chambers (26/22 °C day/night, 16-h photoperiod, 300 μmol m^−2^ s^−1^ PPFD, 50–60% RH). After seedling for 4 d, the soybean plants with 5-cm primary roots were transferred to hydroponic systems containing 0.5 mM CaCl_2_ (pH 4.5) ± 50 μM AlCl_3_. By cultivating for 24 h, the roots were harvested for phenotypic and molecular analyses (*n* = 4 biological replicates, each with 6 plants).

### 4.7. Root Length Measurement

Root length was measured using a ruler at 0 and 24 h. The Al tolerance was evaluated by the relative root growth (RRG) [60]. The RRG can be calculated as follows:

Root elongation (RE) = RL_24h_ − RL_0h_

Relative root growth (RRG) = RE_+Al_/RE_−Al_

### 4.8. Spatial Localization and Quantification of Al Accumulation in Root Tips of Al Accumulation in Root Tips

To visualize Al accumulation in root, hematoxylin staining was performed according to previously described [61] with minor modifications. Briefly, Al-treated roots were washed with 0.5 mM CaCl_2_ for five times to remove surface-bound Al^3+^, then root tips were stained with 0.1% (*w*/*v*) hematoxylin for 30 min, and washed with 0.5 mM CaCl_2_ three times (5 min per rinse). Imaging was conducted immediately after the final rinse to ensure optimal visualization of Al accumulation.

To measure Al concentration, root tips of soybean seedlings were washed with double-distilled water (ddH_2_O) to fully remove the residual Al on the surface. The roots were excised with 0.5 mM CaCl_2_, then rinsed in Ultrafree-MC Centrifugal Filter Units (Millipore) and centrifuged at 5000× *g* for 10 min at 4 °C to remove apoplastic solution. The roots were then frozen at −80 °C overnight. The root cell sap solution was obtained by thawing the samples at room temperature and then centrifuging at 20,600× *g* for 10 min according to Li et al. [62]. Additionally, for total Al concentration, 50 mg fresh root tip samples were digested in 500 μL of concentrated HNO_3_ for 2 d, followed by dilution in 5 mL of 2% (*v*/*v*) HNO_3_. Both of Al in cell sap and root tips were finally determined by inductively coupled plasma atomic emission spectroscopy (ICP-AES) on a 710-ES instrument (Varian, Palo Alto, CA, USA).

### 4.9. RNA Isolation and Transcriptomic Analysis

Root tips treated with 0 (−Al) or 50 (+Al) μM AlCl_3_ for 24 h were used for RNA isolation and transcriptomic analysis, which were conducted by ShenZhen BGI Genomics Co., Ltd. (ShenZhen, China). Total RNA was extracted using TRIzol reagent (Invitrogen, Carlsbad, CA, USA). The quantity and quality of the RNA were measured with a Nanodrop 2000c spectrophotometer (Thermo Fisher Scientific, Waltham, MA, USA) and an Agilent 2100 (Agilent Technologies, Santa Clara, CA, USA). Following quality control, cDNA libraries were prepared using the NEBNext^®^ Ultra^TM^ RNA Library Kit (NEB, Ipswich, MA, USA) and sequenced on an Illumina HiSeq^TM^ 4000 platform (Illumina, San Diego, CA, USA) with 150-bp paired-end reads (PE150). Four biological replicates were performed for each treatment condition.

Raw RNA-seq data were obtained using the Casava v.1.8. The quality of the raw reads was checked with the FastQC package, after which the adaptor, poly-N, and low-quality reads were removed. The obtained clean reads of all the samples were assembled by Trinity software (v2). To annotate the assembled transcripts, BLASTx searches (E-value < 1 × 10^−5^) were performed with the National Center for Biotechnology Information (NCBI) Non-Redundant protein (NR), Swiss-Prot, Kyoto Encyclopedia of Genes and Genomes (KEGG), and Clusters of Eukaryotic Orthologous Groups of Proteins (KOG) databases. The transcript abundance was normalized by the expected number of fragments per kilobase of transcript sequence per million base pairs (FPKM) using the RNA-Seq by Expectation Maximization (RSEM) package. The differentially expressed genes (DEGs) between treatments were identified using the DESeq2 (v1.22.2) with the following significance thresholds: |log2(fold change)| ≥ 1 and an adjusted q-value (FDR) < 0.05. GO (Gene Ontology, http://geneontology.org/) enrichment of DEGs was performed using a hypergeometric test with *q*-value correction [63]. All raw sequencing data have been deposited in the Gene Expression Omnibus (GEO accession number GSE25302369).

### 4.10. Quantitative Real-Time PCR (qRT-PCR) Analysis

First-strand cDNA was synthesized from total RNA with the Revert Aid First Strand cDNA Synthesis Kit (Thermo, Waltham, MA, USA). qRT-PCR analysis was performed using SYBR Green master mix (Vazyme, Nanjing, China) on a QuantStudio™ 6 Flex Real-Time PCR System (Thermo Fisher Scientific, Waltham, MA, USA). The reference gene *GmEF1-α* (*Glyma.17G186600*) was used as the internal control. Gene expression was calculated relative to the expression of the reference gene [42]. qRT-PCR primers are provided in Table S1. Analyses included four biological replicates.

### 4.11. Subcellular Localization of the Soybean HIPP Family Member GmHIPP29

The full-length cDNA was amplified using gene-specific primers *GmHIPP29-GFP-F*/*R* (Table S1) and cloned it into the *pEGAD* vector to generate a C-terminal GFP fusion construct (*35S:GFP-GmHIPP29*) [64]. Both the recombinant vector and empty vector control (*35S:GFP*) were transformed into *Agrobacterium tumefaciens* GV3101 for transient expression in tobacco (*Nicotiana benthamiana*) leaves. The plasma membrane marker plasmid of *AtPIP2A-mCherry* was co-transformed with the 35S:GFP and 35S:GFP-*GmHIPP29* constructs for co-localization analysis. The green fluorescence derived from GFP was observed by the confocal scanning microscope at 488 nm excitation/507 nm emission (Zeiss LSM780, Oberkochen, Germany).

The localization pattern was also performed in rice protoplasts. The recombinant vector *35S:GFP*-*GmHIPP29* and *pEGAD* vector were transiently transformed into rice protoplasts together with *AtPIP2A*-mCherry as a plasma membrane marker [65]. After incubation for 24 h, the transformed protoplasts containing the 35S:GFP-*GmHIPP29* or *35S:GFP* vector were separately treated with 0.25% trypsin (*v*/*v*) for 10 min. After that the GFP and mCherry signals were observed using a laser confocal fluorescence microscope (Zeiss LSM7 DUO, Oberkochen, Germany) at the wavelengths of 488 nm and 568 nm, respectively.

### 4.12. Functional Analysis of GmHIPP29 in Soybean Hairy Roots

The coding region of *GmHIPP29* was amplified using primers *GmHIPP29-*OX-F/R (Table S1) and inserted into the modified *pTF101s* vector to produce a *35S:GmHIPP29* construct. For RNA interference constructs, a 223-bp fragment of its CDS was amplified by gene specific primers listed in Table S1, and inserted into the linearized *pFGC5941* vector driven by *CaMV 35S* in both sense and antisense orientation as previously described [36]. Subsequently, the *GmHIPP29-*OX, *GmHIPP29-*RNAi, and their corresponding empty vectors were separately transformed into *Agrobacterium rhizogenes* strain K599, which was further used to infect soybean cotyledons to obtain transgenic hairy roots as described previously [33,36]. Transgenic hairy roots verified by qRT-PCR assays were used for further analysis.

To evaluate the effects of alternated *GmHIPP29* expression on soybean hairy root growth in response to Al stress, uniform hairy roots were selected, and initial root length was analyzed. Subsequently, hairy roots were subjected to 0 and 50 µM AlCl_3_ in 0.5 mM CaCl_2_ solution (pH 4.5) in a growth chamber with 28 °C for 24 h. After Al treatment, root length was measured. Root elongation (24 h root length–0 h root length) in 50 µM AlCl_3_ was evaluated, Al concentration in the root tip cell sap and root tips were measured. Each treatment had at least 16 independent transgenic hairy roots.

### 4.13. Statistical Analysis

Descriptive statistics (means ± standard error) were calculated using Microsoft Excel 2021 (Microsoft Company, Redmond, WA, USA). Differences between treatments analyzed using Student’s *t*-test (*, *p* < 0.05. **, *p* < 0.01. ***, *p* < 0.001) in SPSS v26 (IBM Corp., Chicago, IL, USA).

## 5. Conclusions

In this study, a comprehensive and systematic analysis of *GmHIPP* family genes was conducted for the first time in soybean. There was an uneven chromosome distribution for 76 *GmHIPP* genes in soybean. Phylogenetic tree and synteny analyses of *GmHIPP* genes among soybean, rice, and Arabidopsis provided new insights into the evolutionary characteristics. The expression patterns of *GmHIPP* family genes in Al-treated soybean were explored, and 20 *GmHIPP* genes were regulated under Al toxicity. In addition, the overexpression of *GmHIPP*29 in transgenic hairy roots reduced the Al tolerance. This study not only systematically and comprehensively described the *GmHIPP* family genes but also screened candidate genes *GmHIPP*29 for functional verification in transgenic hairy roots. These results could be helpful to reveal the molecular mechanisms of Al tolerance in soybean, and used as putative target to breed Al-tolerant soybean cultivars.

## Data Availability

The datasets generated and analyzed during the current study are available from the corresponding author on reasonable request.

## Supplementary Materials

The following supporting information can be downloaded at: https://www.mdpi.com/article/10.3390/plants14233582/s1, Table S1. Primers used for qRT-PCR and PCR analysis. Table S2. The detailed information of GmHIPP genes in soybean. Table S3. DEGs involved in HMA domain-containing protein. Figure S1. Exon-intron architecture of GmHIPPs. The green rectangles and black lines represent exons and introns, respectively. Figure S2. qRT-PCR analysis of representative genes that are differentially expressed according to the transcriptomic results (*p* < 0.05).
