# Supplementary material for: Characterization of the Soybean (Glycine max) Heavy-Metal-Associated Isoprenylated Plant Protein (HIPP) Gene Family in Response to Aluminum
Source: Plants (Basel). 2025 Nov 24;14(23):3582. doi: 10.3390/plants14233582 (PMC12694265; doi:10.3390/plants14233582)
Supplement: Supplementary file 1 [file plants-14-03582-s001.zip › Supplementary Figure.pdf]

Figure. S1

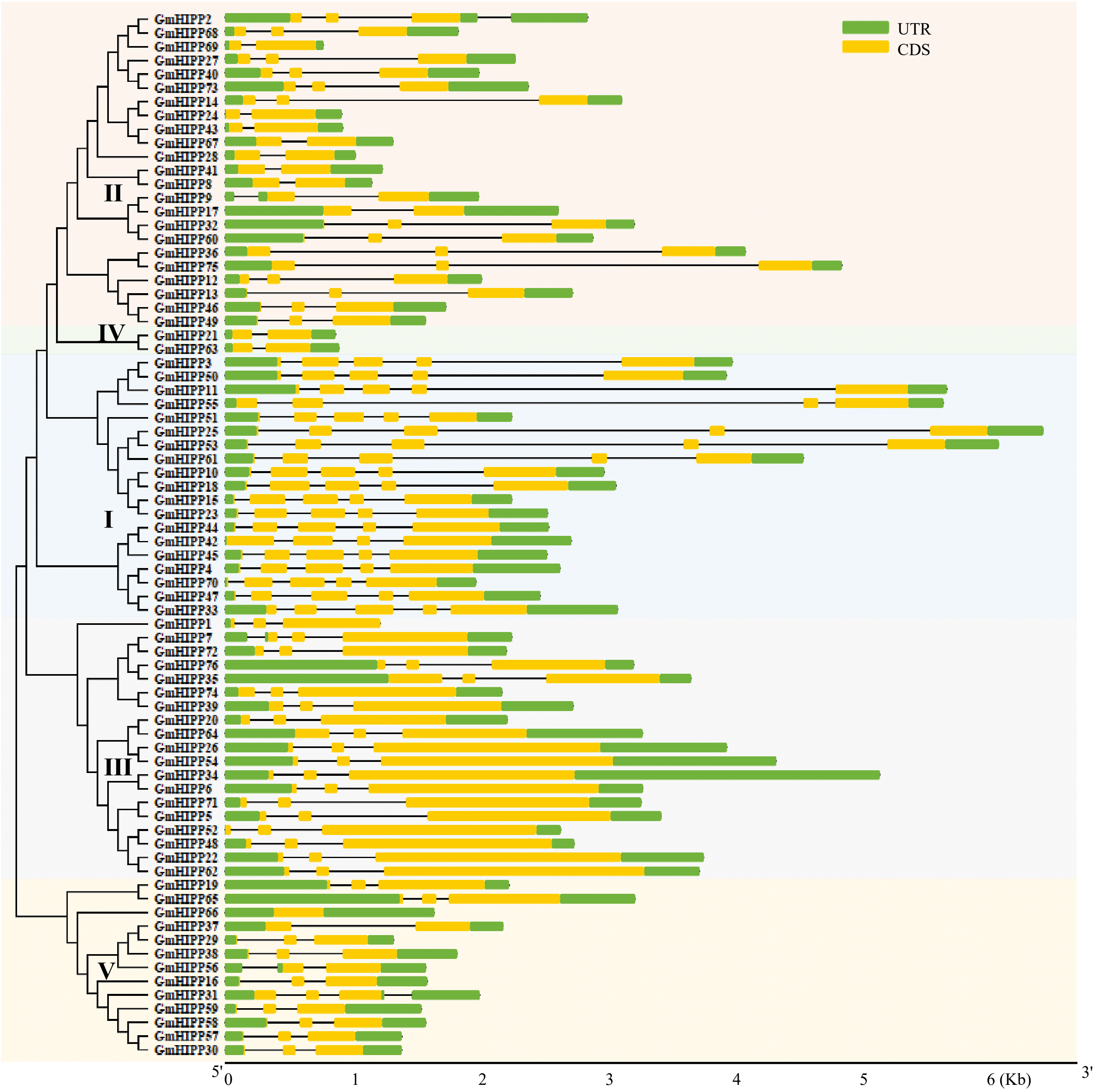

Fig. S1. Exon-intron architecture of *GmHIPPs*. The green rectangles and black lines represent exons and introns, respectively.

Figure. S2

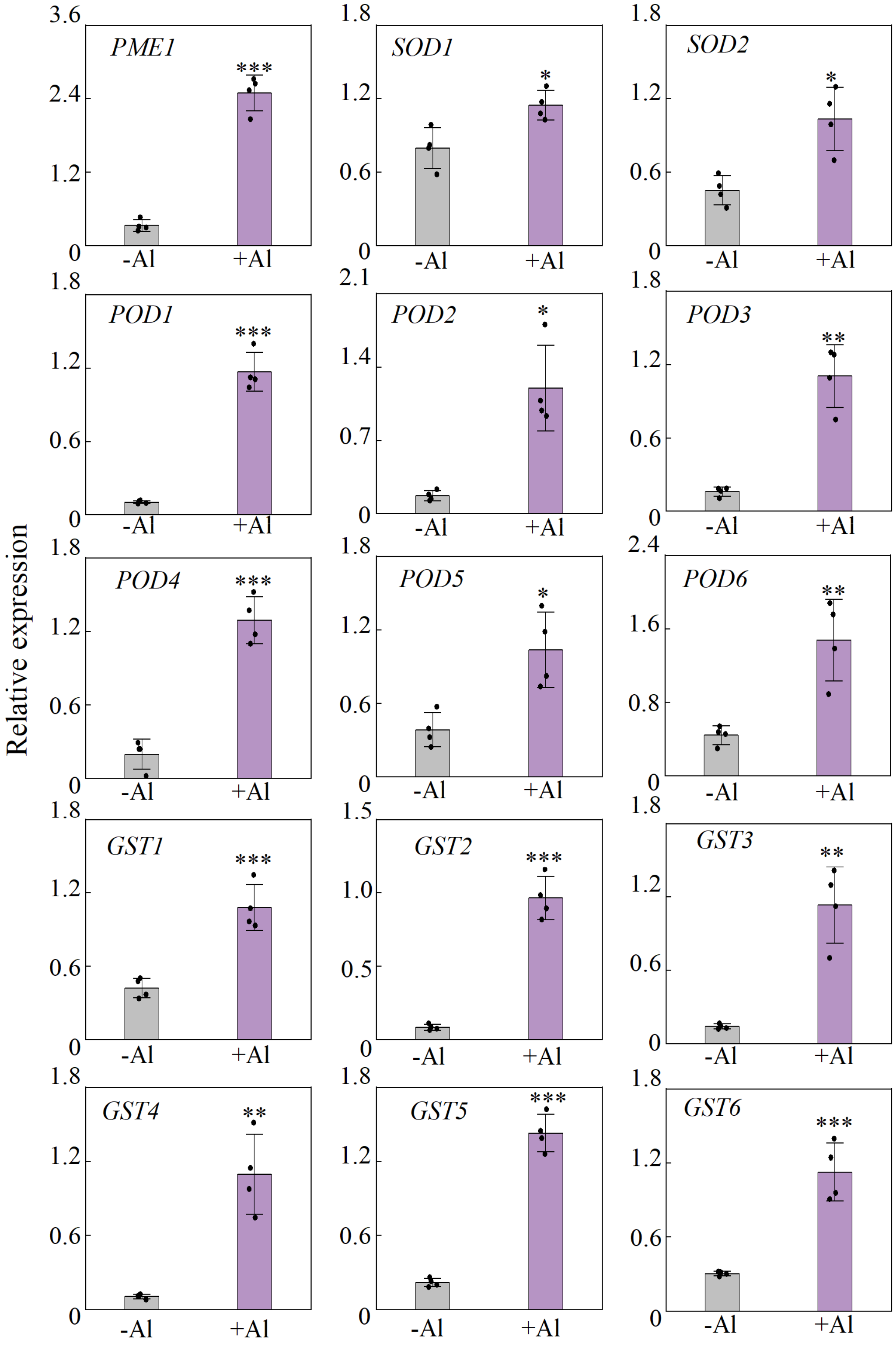

Figure. S2. qRT-PCR analysis of representative genes that are differentially expressed according to the transcriptomic results (P < 0.05). Asterisks indicate significant differences between the +Al treatment and –Al control using Student's t-test. *, P<0.05. **, 0.001<P<0.01. ***, P<0.001.
